# Socio-demographic determinants of cervical cancer screening uptake among women of child-bearing age in Mangochi, Malawi: a facility-based cross-sectional study

**DOI:** 10.1186/s12885-022-10154-w

**Published:** 2022-10-26

**Authors:** Felistas Mpachika-Mfipa, Lucy Ida Kululanga, Abigail Kazembe, Dumisani Mfipa

**Affiliations:** 1 Nursing Department, Phalombe District Health Office, Phalombe, P. O. Box 79, Malawi; 2 School of Nursing, Department of Community Health Nursing, Kamuzu University of Health Sciences, P/Bag 360, Blantyre, Malawi; 3 School of Maternal, Neonatal and Reproductive Health, Department of Midwifery , Kamuzu University of Health Sciences, P/Bag 360, Blantyre, Malawi; 4 Amref Health Africa, Lilongwe 3, P.O. Box 30768, Malawi

**Keywords:** Determinants, Women of child-bearing age, Cervical cancer screening, Uptake

## Abstract

**Background:**

Cervical cancer (CC) is the number one leading cause of death among women in Malawi. However, it is generally viewed as preventable and treatable if diagnosed in an early stage. Despite the burden, Malawi registers low uptake of cervical cancer screening (CCS). This study examined the socio-demographic determinants of CCS uptake among women of child-bearing age (WCBA) in Mangochi district.

**Methods:**

A cross-sectional quantitative study was conducted in five health facilities. A total of 482 women between the ages of 18–49 participated and were sampled using a multi-stage sampling method. An interviewer administered structured questionnaire was used to collect data from June to July, 2019. Multivariate logistic regression model was used to identify determinants of CCS uptake among WCBA.

**Results:**

Few respondents (13.1%) had ever done CCS. Compared to respondents in the age-group of 18–24 years, those in the age-groups of 25–35 years and 36–49 years were 2.63 and 3.90 times more likely to undergo CCS (AOR = 2.63, 95% CI 1.30–5.31 and AOR = 3.90, 95% CI 1.62–9.38), respectively. Respondents who practiced Christianity were 2.77 times more likely to undergo CCS than those who practiced Islam (AOR = 2.77, 95% CI 1.23–6.22). Respondents of the Chewa ethnic group were 71% less likely to undergo CCS as compared to those of Yao ethnicity (AOR = 0.29, 95% CI 0.09–0.95). Respondents who lived in semi-urban areas were 2.57 times more likely to go for CCS than those who were village residents (AOR = 2.57, 95% CI 1.19–5.55).

**Conclusion:**

Our study showed that CCS uptake was low in Mangochi and the results suggested that age, religion, ethnicity and place of residence were determinants of CCS uptake. We recommend that comprehensive health education on CC should specifically target the young women and Muslim women in places where they meet. We call upon the district health authorities to scale up CCS provision in all Antiretroviral Therapy (ART) and outreach clinics to improve CCS uptake among women residing in the villages and those of the Chewa ethnicity. We, further, call upon all CC program implementers to design programs that address the highlighted socio-demographic determinants of CCS uptake among WCBA in the district.

## Background

Cervical cancer (CC) is one of the preventable reproductive cancers that cause high morbidity and mortality in women [1]. Over 1 million women worldwide are estimated of having CC, with most of the cases being undiagnosed [1]. In 2020, globally, 604,127 women were newly diagnosed of having CC and 341,831 women were estimated to have died in the same year [2]. The burden of CC is equally high in Southern Africa where the CC age standardized incidence rate is at 36.4 per 100,000 women annually with a very high age standardized mortality rate at 20.6 per 100,000 women [2]. Developing countries have a higher burden of CC when compared with the developed ones [3]. In the United States of America, the CC incidence rate is reported to be at 6.2/100,000 women [2]. In comparison, Zambia’s CC incidence rate is estimated at 65.5/100,000 women [2]. In Malawi, CC is the number one leading cause of death among women [4] with the CC age standardized rate (ASR) of 67.9/100,000 women [2]. It is estimated that 4,145 women develop CC and 2,905 die from the disease every year in Malawi [2]. CC prevention and treatment are effective when diagnosed early and managed effectively [1]. WHO recommends the screen and treat approach to be made available and accessible to the largest proportion of women at risk even to those in low-income countries [1]. Visual Inspection with Acetic Acid (VIA) is one of the cheap and effective means of screening for precancerous lesions which Malawi adopted in 2005 [5]. The causes of poor cancer survival rates in the developing countries have been widely reported and they include ineffective screening programs, low cancer awareness among the general public and healthcare workers, a shortage of qualified professionals, oncology personnel, treatment facilities, and equipment that lead to late presentation, and advanced CC is frequently diagnosed [6–8]. Findings from a Nigerian study suggested that fear of positive results, lack of test awareness, reluctance to screen, low-risk perception, and lack of time were major barriers to CCS [9]. A Malawian study done in Blantyre district found low uptake of CCS estimated at 13.2% [10]. Some of the factors that had contributed to this low uptake of the screening services were lack of knowledge among women on CCS, unavailability of services in some health facilities, gender and age of the health worker providing the screening services [3, 10–12]. CCS remains a challenge in Mangochi district in Malawi. The district’s CC annual report showed that only 2.9% of women of child-bearing age (WCBA) were screened in 2019 [13]. Despite the high burden of CC in Malawi, the CCS rate is very low as only 30.6% of women had been screened in the country [5].

Globally, CCS has been identified as an equity issue, where socio-demographic and economic factors affect uptake of CCS [14]. The act of screening can also be influenced by other factors such as knowledge, health beliefs and health system. An individual’s knowledge and health beliefs about the importance or the magnitude of a health problem may or may not lead to a decision to seek medical care [15]. We applied Andersen’s Behavioural Model of Health Services Use which highlights contextual and individual determinants to improve access to healthcare services [15]. Specifically, the Model explains or predicts one’s use of healthcare services by focussing on an individual’s predisposition to use healthcare services, enabling factors that facilitate or impede use of healthcare services, and one’s perceived or influenced need for care [15]. However, this is a preliminary study that assessed the socio-demographic factors (predisposing factors) associated with uptake of CCS services in Mangochi district, Malawi. Further assessments of other factors (individual perceptions, beliefs and health system) will be conducted in the next paper.

## Materials and methods

### Study design and population

This was a cross-sectional study that utilised a quantitative approach and was conducted in selected health facilities in Mangochi district from June to July 2019 with an aim of identifying determinants of CCS among WCBA. The district covers an area of 6,273 square kilometres in the southern region of Malawi and it shares boundaries with Balaka, Machinga, Ntcheu, and Salima districts and also an international border with Mozambique. The majority of people in the district belong to the Yao ethnic group but Chichewa and Chiyao languages are commonly used across the district. Fishing, farming and small scale businesses are the main sources of income. According to Malawi National Statistical Office (NSO) population projections for 2016/2017, the district had a total estimated population of 1,053,585 and out of that 242, 325 were WCBA. The estimated population of the area under study was 185,371 with 23% (42,635) being WCBA [16]. The study population included all WCBA (18–49 years old).

## Study sites

**Fig. 1:**
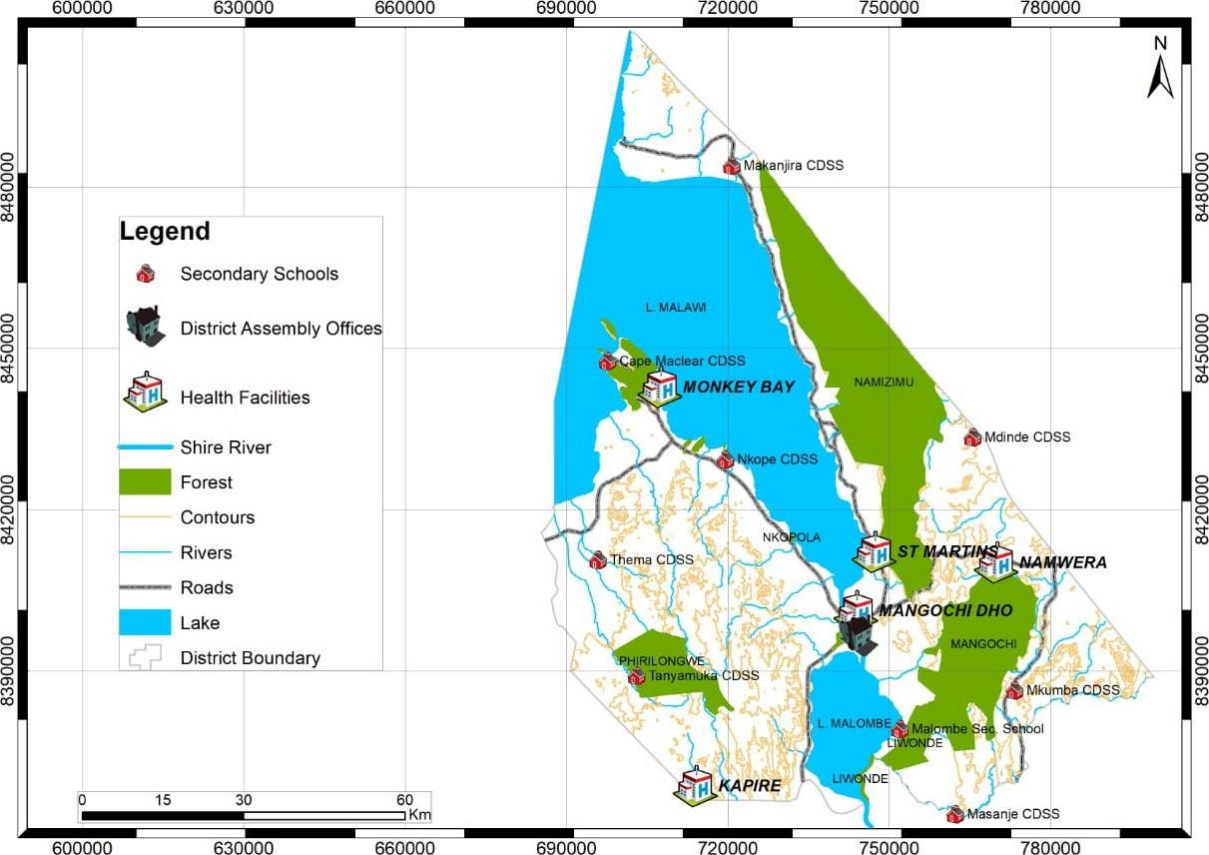
Map of study sites in Mangochi district

Mangochi district has five zones namely; Mangochi Boma, Monkey-Bay, Chilipa, Namwera and Makanjira. From each zone, one health facility offering CCS was randomly selected as a study site. Thus, a total of five health facilities from 14 health facilities offering CCS namely; Mangochi District Hospital, Monkey-Bay Community Hospital, St. Martin’s Community Hospital, Kapire Health Centre and Namwera Health Centre were the study sites (Fig. 1).

## Sample and procedures

The sample size was derived using the Cochran formula 1977 [17]. With the prevalence of CCS in Malawi being at 30.6% [18] and Z = 1.96 at 95% Confidence Interval (CI) and a precision of 5%, a design effect of 1.4 and a non-response rate of 10%, a sample size of 502 was obtained. However, the actual sample size whose data was analysed was 482 (there was a 96% response rate and 4% of the respondents opted out).

A multistage sampling technique was used to identify the study respondents. Health facilities were simple randomly selected from each zone using a ballot box and individual respondents were again simple randomly picked through balloting to participate. The total number of respondents to be drawn from each facility was allocated using the proportionate sample allocation formula [19]. The sample size for each health facility was as follows: Monkey-Bay community hospital, 105; Mangochi district hospital, 209; St. Martin’s community hospital, 91; Kapire health centre, 30 and Namwera health centre, 47.

To recruit the respondents, a brief description of the study was made each morning in the above mentioned facilities to have the clients informed of a possibility of being approached to participate. Female clients sitting on the queues waiting for consultation were then provided with numbers on a piece of paper to be used for random sampling. Nurses and clinicians working in these areas (who had prior orientation of the study) had similar numbers as those with clients put in a box and they randomly picked the numbers and sampled the clients. Those WCBA who were less than 18 years or older than 49 years, and were accessing In-Patient services, including those who were very sick and those who had refused to take part in the study were excluded from going to the recruitment room. Whilst those WCBA aged between 18 and 49 years, who were accessing Out Patient Department services, and were in a stable condition and had accepted to participate in the study met the inclusion criteria and were at the end of the consultation had an assigned support staff to escort them to a recruitment room. Those clients who were at the far end of the consultation line, and their numbers had been selected during balloting, were first approached by the research assistant to be recruited to minimize the time the clients spent for both consultation and study participation. In the recruitment room, research assistant gave thorough information of the research and allowed the client to give an informed consent. Once consent was given, the client was recruited as a study participant. To ensure anonymity, respondents’ names were not included on the questionnaire, instead, codes were used to identify the respondents. The information that the respondents provided was only shared with the research team to maintain confidentiality and the respondents were voluntarily asked to participate in the study and drop out at any point without any repercussions or affecting the other health services that they had come to access. Data was collected from June to July, 2019.

## Questionnaire, independent and dependent variables

Data was collected from WCBA who were accessing out-patient department services such as family planning clinic, antiretroviral therapy clinic and antenatal clinic from the five selected health facilities using a structured questionnaire. The questionnaire was adapted with permission of the author from a study conducted by [20]. This data collection tool was not a standardised tool, however, it was pretested and used in the initial study which was later peer reviewed and published. The questionnaire was translated to Chichewa and Chiyao languages and back to English to compare with original text, the two local languages are commonly used in the study areas. Experts in English, Chichewa and Chiyao languages as well as CC field checked the tools to ensure that the originality of the tool was not lost. The questionnaire was pre-tested at a health centre that was not part of the actual study to check for clarity of the questions and correct any errors on the tool. A trained research assistant (a registered nurse midwife) administered the questionnaire to the respondents. The following were the questions which were asked under each variable; what is your age? What is your religion? What is your highest level of education? What is your ethnicity? What is your marital status? If you are married, what is the relationship type? Where do you reside? What do you do to earn a living? And, have you ever been screened for cervical cancer? In Malawi, CCS is a free sexual and reproductive health and rights (SRHR) service in both public and faith-based owned health facilities from which the study sample was drawn from as such study participants did not pay direct (out of pocket) costs for the service. However, participants incurred transport cost from home to the health facilities and the cost varied depending on the distance. Transport cost was not analysed in the current study as a factor associated with uptake of CCS. Of the analysed variables, data on age was collected as a continuous variable and categorised as and coded into ranges of 18–24 (1), 25–35 (2) and 36–49 (3) years old. Religion was categorised and coded as Islam (1) and Christianity (2). Educational status was categorised and coded as primary (1), none (2), secondary (3) and tertiary (4). Ethnicity was categorised and coded as Yao (1), Chewa (2), Lomwe (3) and Other (4). Marital status was categorised and coded as married (1), single (2), widowed (3) and divorced/separated (4). Relationship type was categorised and coded as monogamous (1) and polygamous (2). Occupation was categorised and coded as housewife (1), business (2), farming (3) and the employed (4). Residence was categorised and coded as village (1), trading centre (2) and semi-urban area (Boma) (3). The outcome variable of whether respondents had ever undergone for CCS had a binary response (yes = 1 and no = 0).

### Statistical analysis

Descriptive statistics of the socio-demographic characteristics, including the proportion of WCBA between 18 and 49 years old that had undergone CCS were calculated. Bivariate logistic regressions were conducted to examine socio-demographic factors significantly associated with CCS uptake among WCBA. Those factors significantly associated with CCS uptake among WCBA at bivariate level were included in the multivariable logistic regression model. The results of bivariate and multivariable analyses have been reported as crude odds ratios (CORs) and adjusted odds ratios (AORs) with 95% CIs, respectively. A *p*-value of less than 0.05 was considered statistically significant in this study. Data were entered and analysed using an IBM SPSS Statistics for Windows Version 20.0 (IBM Corp., Armonk, NY, USA).

## Ethical considerations

This study was approved by the Malawi College of Medicine [now Kamuzu University of Health Sciences – KUHeS] Research and Ethics Committee (COMREC) with an approval number of P.04/19/2646 and was performed according to the Declaration of Helsinki. The informed consent for participants who could not read and write was approved by COMREC along with the main study protocol. A clearance was sought from Mangochi District Health Research Committee and health facilities before engaging the participants. All participants provided written informed consent prior to participation – Literate participants read and signed the informed consent form whereas those who could not read and write had the informed consent read to them and marked with a thumb print as evidence of consent.

## Results

### Socio-demographic characteristics of respondents

A total of 482 women participated in the study. Less than quarter (13.1%) had ever been screened for CC, 42.5% were of ages from 18 to 24 years, 66.0% were Muslims and 64.1% belonged to the Yao tribe. Slightly below two-thirds (64.9%) of the respondents had primary education as their highest qualification and more than three-quarters (86.7%) of the respondents were married of which 79.7% (333/418) of them were in a monogamous relationship. Slightly over one-third (34.4%) of the respondents were housewives and more than two-thirds (69.5%) lived in villages (Table 1).

**Table 1:** Socio-demographic characteristics of respondents in Mangochi, Malawi

WCBA’s Characteristics	N = 482 n (%)	Ever screened N = 63, n (%)
Age-group (years)		
18–24	205 (42.5)	13 (20.6)
25–35	208 (43.2)	35 (55.6)
36–49	69 (14.3)	15 (23.8)
Religion		
Islam	318 (66.0)	28 (44.4)
Christianity	164 ( 34.0)	35 (55.6)
Educational status		
None	87 (18.0)	12 (19.1)
Primary	313 (65.0)	37 (58.7)
Secondary	78 (16.2)	13 (20.6)
Tertiary	4 (0.8)	1 (1.6)
Ethnicity		
Yao	309 (64.1)	31 (49.2)
Chewa	75 (15.6)	5 (8.0)
Lomwe	48 (9.9)	14 (22.2)
Other	50 (10.4)	13 (20.6)
Marital status		
Married	418 (86.7)	53 (84.1)
Single	24 (5.0)	1 (1.6)
Widowed	8 (1.7)	4 (6.3)
Divorced/Separated	32 (6.6)	5 (8.0)
Occupational status		
Housewife	166 (34.4)	20 (31.8)
Business	128 (26.6)	15 (23.8)
Farming	127 (26.3)	20 (31.8)
Employed	61 (12.7)	8 (12.6)
Place of residence		
Village	335 (69.5)	34 (54.0)
Trading Centre	89 (18.5)	14 (22.2)
Semi-urban (Boma)	58 (12.0)	15 (23.8)
Relationship Type	**N = 418**	** N = 53**
Monogamous	333 (79.7)	44 (83.0)
Polygamous	85 (20.3)	9 (17.0)

## Socio-demographic factors associated with CCS uptake among WCBA respondents in Mangochi, Malawi

Compared to respondents in the age-group of 18–24 years, those in the age-groups of 25–35 years and 36–49 years were 2.63 and 3.90 times more likely to undergo CCS (AOR = 2.63, 95% CI 1.30–5.31 and AOR = 3.90, 95% CI 1.62–9.38), respectively. Respondents who practiced Christianity were 2.77 times more likely to undergo CCS compared to those who practiced Islam (AOR = 2.77, 95% CI 1.23–6.22). Respondents of the Chewa ethnic tribe were 71% less likely to undergo CCS than those of the Yao ethnic tribe (AOR = 0.29, 95% CI 0.09–0.95). Respondents who lived in semi-urban area (Boma) were 2.77 times more likely to undergo CCS as compared to those living in the village (AOR = 2.57, 95% CI 1.19–5.55) (Table 2).

**Table 2:** Socio-demographic factors associated with CCS uptake among WCBA respondents in Mangochi, Malawi

Factors	COR (95% CI)	AOR (95% CI)	P-value
Age-group			
18–24	Reference	Reference	
25–35	2.99 (1.53–5.83)*	2.63 (1.30–5.31)*	0.007
36–49	4.10 (1.84–9.15)*	3.90 (1.62–9.38)*	0.002
Religion			
Islam	Reference	Reference	
Christianity	2.81 (1.64–4.82)*	2.77 (1.23–6.22)*	0.014
Ethnicity			
Yao	Reference	Reference	
Chewa	0.64 (0.24–1.71)	0.29 (0.09–0.95)*	0.041
Lomwe	3.69 (1.79–7.62)*	1.55 (0.60–4.01)	0.366
Other	3.15 (1.51–6.56)*	1.25 (0.47–3.32)	0.652
Marital Status			
Married	Reference	Reference	
Single	0.30 (0.04–2.26)	0.51 (0.06–4.23)	0.531
Widowed	6.89 (1.67–28.37)*	3.95 (0.86–18.15)	0.077
Divorced/Separated	1.28 (0.47–3.46)	1.29 (0.45–3.73)	0.632
Residence			
Village	Reference	Reference	
Trading centre	1.65 (0.84–3.24)	1.31 (0.63–2.72)	0.473
Semi-urban (Boma)	3.09 (1.56–6.14)*	2.57 (1.19–5.55)*	0.016

*p ˂ 0.05

Multivariate model adjusted for age-group, religion, ethnicity, marital status and area of residence.

**Note:** Educational status, relationship type and occupation were not statistically significant at bivariate level.

## Discussion

This study was conducted to determine socio-demographic factors that are associated with CCS uptake in Mangochi, Malawi. Our findings showed that uptake of CCS in Mangochi is low. This finding is consistent with other studies done in Malawi and across Africa [10, 21–24]. Malawi government is still scaling up the number of health facilities and providers of CCS and also currently the integration of HIV care and CCS has been introduced in some facilities in the districts. These interventions might improve the uptake of screening in the years to come. On the other hand, the Mangochi district CC program report for 2019 showed that 2.9% of WCBA had been screened [13]. This study, however, found that 13.1% of the respondents had ever been screened. This finding can be attributed to the nature of the study question as it was asking whether the respondent had ever screened for CC and not specifically only those who had screened in 2019. It could be possible that most of the respondents had screened for CC in previous years. Our study suggested that older women in the age-groups of 25–35 years and 36–49 years were more likely to undergo CCS as compared to adolescent girls and young women (AGYW) in the age-group of 18–24 years. This result could be linked to the setting where these participants were found as most young people are generally healthy and do not visit health facilities where information and services on CC are mostly provided. This finding could also be attributed to the Ministry of Health age stipulation for those women that are eligible for CCS in Malawi. According to the National Service Delivery Guidelines for CC Prevention and Control, women in the 25–49 years age-group and only sexually active women aged 21–25 are eligible for CCS using VIA in Malawi [4]. This age eligibility criterion in the national guidelines could prevent health workers from advocating, educating and offering CCS to young women less than 25 years of age. This current study included women less than 25 years old because in HIV positive women, the progression from precancerous lesion to cancer is much faster than in HIV negative women [1]. By 2018, the Malawi Ministry of health had started integrating CCS in HIV care. This finding on age is not unique and has been reported in other studies [10, 11, 24]. Our study suggested that respondents who were Christians were more likely to undergo CCS as compared to those who were Muslims. This is in agreement with what was found in a Nigerian study that reported a significant association between religion and uptake of Pap smear [25]. On the contrary, several studies have shown that there is no significant association between religion and uptake of CCS [10–12, 22, 24, 26–28]. Mangochi is a predominantly Islam district and there is need to further explore through qualitative research on how religion contribute to the findings in the current study. This study suggested that respondents of the Chewa ethnic group were less likely to undergo CCS as compared to those of the Yao ethnic group. To the best of our knowledge, no research had highlighted a significant association between ethnicity and CCS uptake among similar studies that were reviewed during this write-up. For example, a Nigerian study found no significant association between ethnicity and uptake of Pap smear [25]. Our study finding, therefore, suggests that ethnicity is new knowledge added to a body of literature as a predictor of CCS uptake. We, however, recommend that qualitative research should be conducted to further explore its contribution towards CCS uptake among the study population. We further established that place of residence was a predictor of CCS uptake as semi-urban residents were more likely to be screened than village residents. This result is in agreement with what [28] and [29] found in their studies. We attribute this finding to the availability of screening campaigns and daily VIA clinics at the district hospital, a scenario that is not common in health facilities in the rural areas. A Tanzanian study done by [30] supports this notion as women residing in urban areas were found to be more likely to be knowledgeable about CC than their counterparts who lived in the rural areas. They had affirmed that women in the urban areas had more access to health information through media and they had easy access to the health facilities. We, therefore, recommend that policy makers and health workers should consider raising CCS awareness campaigns, screening campaigns and VIA clinics at health facilities to increase CCS services in the rural areas.

This study had a sample that was large enough and was rigorously sampled from all zones of the district and therefore it was a fair representation of the population of WCBA in Mangochi. However, the study had limitations as it had respondents of ages 18 to 49 only, this may limit the use of the results in women younger than 18 and older than 49. Again, the recruited respondents were those women who were already found in the sampled health facilities. These women’s views may be different from the women who were in the community and were not part of this study, hence the results should be interpreted and be applied with caution. An interviewer administered questionnaire was used to collect data. This may have resulted in recall bias by some respondents and could have an effect on the results of the study. Further, this was a cross-sectional study as such it could not establish the causal-effect relationship between the socio-demographic factors and CCS uptake. These limitations, notwithstanding, this study revealed the determinants of CCS uptake in the study area which can be generalised in a setting similar to where this study was conducted.

## Conclusion

This was a quantitative cross-sectional study that examined socio-demographic determinants of CCS uptake among WCBA in Mangochi, Malawi. The results have shown that uptake of screening is very low at 13.1%. The determinants of CCS in the district include age of the woman, religion, ethnicity and place of residence. We recommend that the age eligible for screening should include all WCBA in this era of HIV infection as HIV infection is a risk factor for CC. We, further, suggest that comprehensive health education should specifically target the young women and Muslim women in places where they meet. In addition, health facilities should include CCS services in their outreach clinics (community-based cervical screening services) to improve CCS uptake among women residing in the villages and those of the Chewa ethnicity. We also recommend that qualitative research should be conducted to adequately understand the influence of religion and ethnicity on uptake of CCS among the study population.

## Data Availability

The datasets generated during and/or analysed during the current study are available from the corresponding author on a reasonable request.

## List of abbreviations

AGYW: Adolescent Girls and Young Women
AORs: Adjusted Odds Ratios
ART: Antiretroviral therapy
CC: Cervical Cancer
CCS: Cervical Cancer Screening
CI: Confidence Interval
COMREC: College of Medicine Research and Ethics Committee
CORs: Crude Odds Ratios
DHO: District Health Office
HIV: Human Immuno-deficiency Virus
SRHR: Sexual and Reproductive Health and Rights
VIA: Visual Inspection using Acetic Acid
WBCA: Women of Child-Bearing Age
